# Case Report: Trastuzumab deruxtecan plus toripalimab in ERBB2-amplified cervical mucosal melanoma

**DOI:** 10.3389/fonc.2025.1625521

**Published:** 2025-09-03

**Authors:** Xiao-Xia Wang, Min-Xin Wang, Chao Sui

**Affiliations:** ^1^ Department of Oncology, Weihai Central Hospital Affiliated to Qingdao University, Weihai, Shandong, China; ^2^ Department of Ultrasound, Weihai Central Hospital Affiliated to Qingdao University, Weihai, Shandong, China

**Keywords:** cervical mucosal melanoma, trastuzumab deruxtecan (T-DXd), genomic profiling, toripalimab, HER2-positive tumors

## Abstract

Cervical mucosal melanoma is a rare, aggressive malignancy with poor prognosis due to delayed diagnosis and limited treatment efficacy. Current therapies, including immune checkpoint inhibitors (e.g., anti-PD-1 agents), chemotherapy, and targeted therapies, yield suboptimal response rates (<30%) and frequent resistance. Here, we report the first case of HER2-positive cervical mucosal melanoma successfully treated with T-DXd combined with Toripalimab. This combination induced significant tumor shrinkage (partial response) and demonstrated safety, highlighting the potential of ADC-based cross-tumor therapies and immune-targeted synergy. These findings support further clinical trials to validate this strategy in mucosal melanoma, addressing unmet needs in this refractory subtype.

## Introduction

Cervical mucosal melanoma is a highly aggressive and rare malignant tumor, accounting for 1%-2% of all melanomas (1). Its biological behavior significantly differs from that of skin or extremity melanomas, exhibiting more pronounced local invasiveness and poorer prognosis (2). Due to its unique anatomical location and lack of early symptoms, most patients are diagnosed at advanced or locally unresectable stages. Currently, standard treatments for unresectable mucosal melanoma include immune checkpoint inhibitors (e.g., anti-PD-1 monoclonal antibodies), chemotherapy, and targeted therapy; however, the overall efficacy remains limited, with an objective response rate of less than 30%, and rapid progression is common (3). In recent years, Toripalimab, a domestic PD-1 inhibitor, has shown promise in metastatic mucosal melanoma (4). Phase II clinical trials have demonstrated that Toripalimab combined with Axitinib as neoadjuvant therapy induces an objective response rate of 48.3%, significantly increasing tumor-infiltrating CD3^+^ and CD8^+^ T lymphocytes, suggesting that immune microenvironment reprogramming may enhance antitumor effects (5). Additionally, in the adjuvant setting, Toripalimab has been shown to significantly prolong relapse-free survival compared to high-dose interferon-α2b and has better tolerability (6). However, monotherapy still faces challenges of primary or secondary resistance, highlighting the need for new combined therapeutic strategies.

Trastuzumab Deruxtecan (T-DXd) is an antibody-drug conjugate (ADC) targeting HER2, which exerts a “bystander effect” via its topoisomerase I inhibitor payload and has demonstrated significant activity in HER2-low expressing tumors (7). Although T-DXd has been approved for use in breast cancer, non-small cell lung cancer, gastric cancer, and gynecologic tumors (8, 9), its application in cervical mucosal melanoma has not been reported. T-DXd selectively targets HER2-positive tumor cells and induces cell lysis, leading to the release of tumor-associated antigens and remodeling of the tumor microenvironment, thereby enhancing tumor immunogenicity and facilitating antigen presentation (10). Meanwhile, toripalimab, a humanized monoclonal antibody against PD-1, blocks the PD-1/PD-L1 axis, thereby restoring T-cell function and promoting antitumor immune responses (11). Preclinical and early-phase clinical studies have demonstrated that the combination of these agents exhibits a favorable safety profile and promising antitumor activity across various solid malignancies (12, 13). Their complementary mechanisms of action provide a strong rationale for use in patients with HER2-positive tumors and an immunologically active tumor microenvironment. Based on genetic profiling that identified HER2 overexpression and/or amplification/mutation in the tumor, this study reports the first successful case of treating HER2-positive cervical mucosal malignant melanoma with T-DXd in combination with Toripalimab. This approach offers a new treatment direction for this refractory subtype. The significant tumor shrinkage (partial response) and safety data not only validate the feasibility of applying ADCs across tumor types but also lay an important foundation for future clinical trials exploring targeted-immune combination therapies in mucosal melanoma.

## Case report

**Figure 1:**
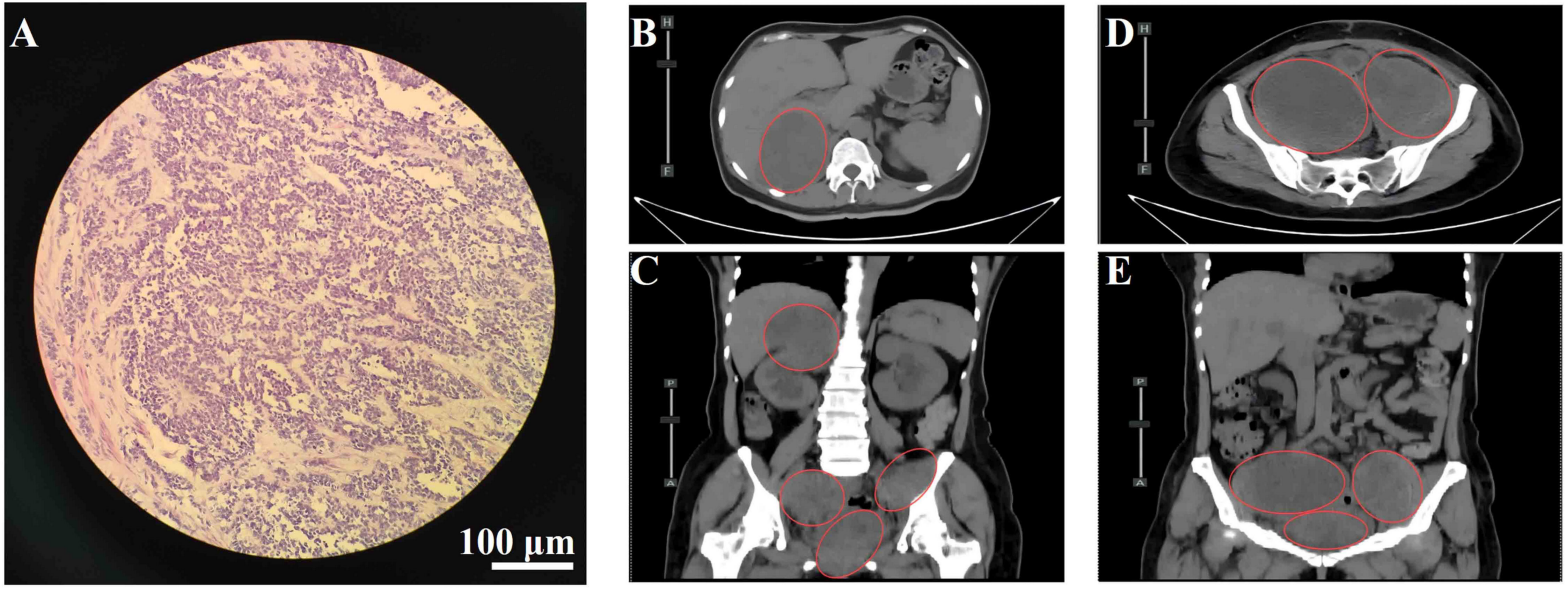
**(A)** Hematoxylin and eosin (H&E) staining of cervical tissue showing malignant melanoma. Scale bar = 100 μm; magnification, 200×. **(B)** Axial view of lesion in the hepatorenal space. **(C)** Coronal view of the hepatorenal space lesion. **(D)** Axial view of pelvic lesion. **(E)** Coronal view of the pelvic lesion. Red circles indicate metastatic lesions.

The patient is a 54-year-old female who presented with a four-month history of a cervical mass, discovered on routine gynecological examination on September 14, 2023. Physical examination revealed a 4 × 3 × 2 cm red, papillary, lobulated mass located on the anterior lip of the cervix. The lesion had a smooth surface and exhibited slight hemorrhagic changes. On September 15, 2023, a hysteroscopic examination was performed, and pathology showed proliferative endometrium in the uterine cavity (**Figure 1A**). The immunohistochemical results show AE1/AE3 (−), CD20 (−), CD3 (−), CD56 (partially +), CD10 (partially +), ER (−), CEA (−), CgA (−), Desmin (−), P16 (−), Ki-67 (index 80%), PR (−), p53 (wild-type), S-100 (+), Syn (−), SMA (−), TTF-1 (−), with scattered positivity for Melan-A and HMB45, and diffuse positivity for SOX10. Based on the histopathological and immunohistochemical findings, a diagnosis of primary malignant melanoma of the cervical mucosa was rendered.

On January 8, 2024, a PET-CT scan was performed, revealing a cervical mass with soft tissue density and increased metabolic activity, consistent with high metabolic activity and suggestive of malignancy. A small lymph node along the right iliac vessel showed mild metabolic increase, indicating possible metastasis, and was recommended for follow-up. Additionally, a small ground-glass nodule in the left upper lobe of the lung exhibited no abnormal metabolic activity, likely representing a non-specific lesion, and was also recommended for follow-up. The scan also identified multiple small pulmonary nodules, likely inflammatory in nature, as well as liver cysts and multiple cysts in the ovaries. There was minimal pelvic fluid present, alongside a small area of softening in the left basal ganglia, and degenerative changes in the spine were noted.

On January 17, 2024, the patient underwent a radical hysterectomy, bilateral adnexectomy, and pelvic lymph node dissection under general anesthesia. Pathology confirmed cervical malignant melanoma (pT4aN0M0, stage IIB). The tumor was located in the cervical canal, measuring 3 × 3 cm, and invaded nearly the full thickness of the cervix. There were no signs of vaginal wall involvement, and the lymph nodes (left 8, right 6) were negative for metastasis. Immunohistochemistry was positive for Vimentin, S-100, SOX10, HMB45, Melan-A, and Ki-67 (80%+), with negative markers for CD45 and CK(P). No adjuvant therapy was administered postoperatively due to the patient’s refusal. The patient was readmitted on August 12, 2024, due to worsening bilateral leg pain for 3 weeks, which had intensified in the past week. On August 14, 2024, an abdominal CT revealed multiple metastatic lesions, including a low-density mass at the liver’s right lobe boundary measuring 3.3 × 3.5 cm, masses in the retroperitoneal space, right kidney, and pelvis with significant tumor invasion near the bladder and rectum, and bone destruction in the T10 vertebra, suspected to be metastatic (**Figures 1B–E**). These findings were consistent with disease recurrence, now classified as cT4aN0M1c, stage IV, with metastases to the bladder, rectum, liver, lungs, bones, abdomen, and pelvis.

**Figure 2:**
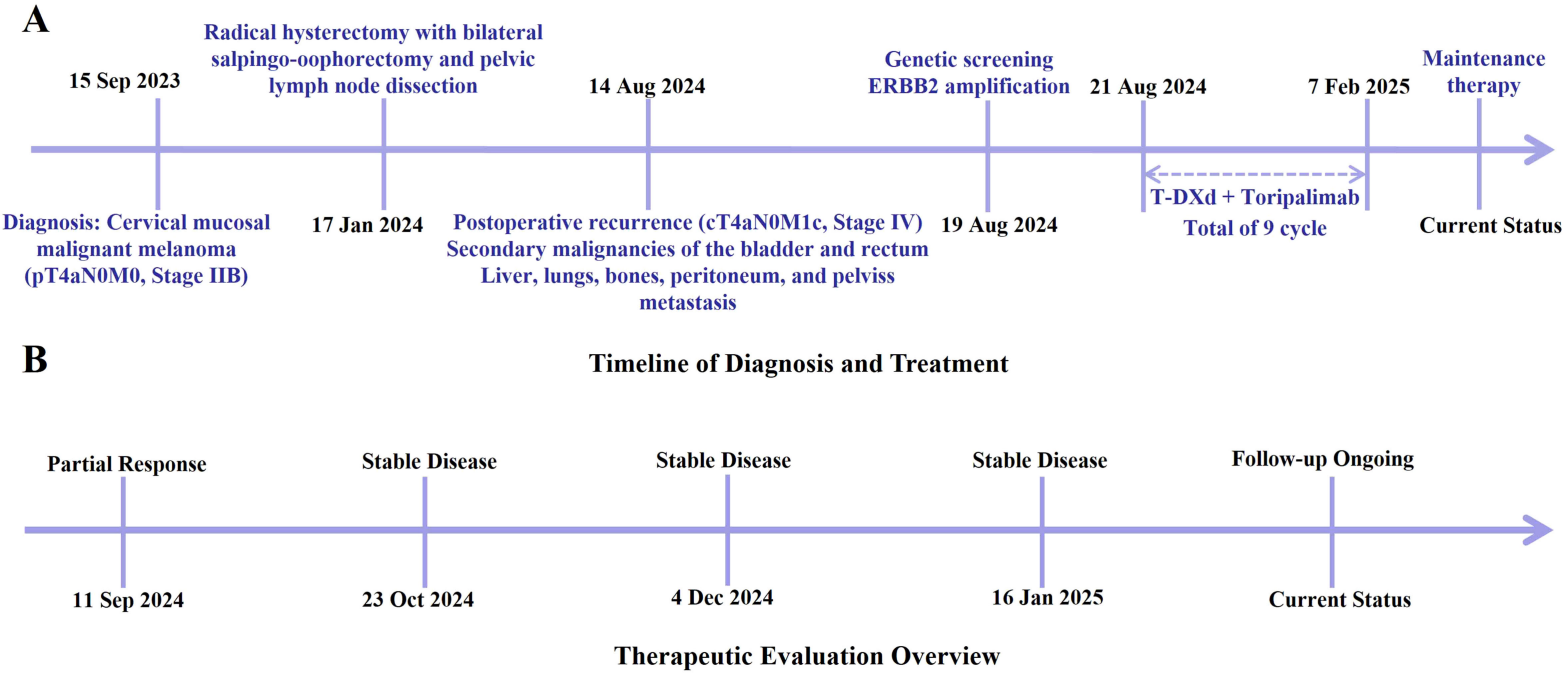
**(A)** Major clinical events from initial diagnosis through the treatment process of this case. **(B)** Overview of the therapeutic response and follow-up timeline.

**Figure 3:**
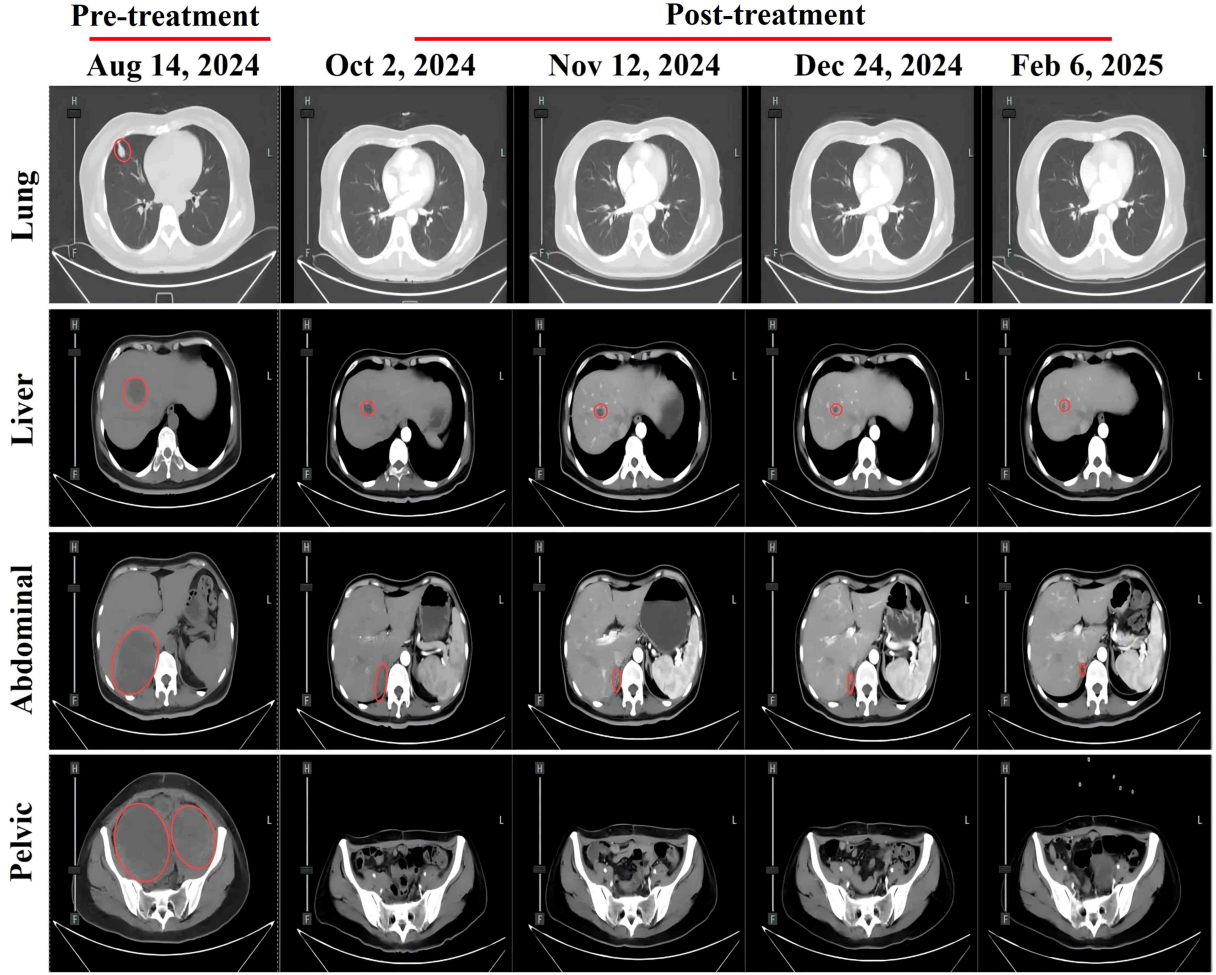
Abdominal CT scans at different treatment time points showing changes in metastatic lesions: pulmonary metastasis, hepatic metastasis, abdominal metastasis, and pelvic metastasis. Red circles indicate metastatic lesions.

The patient developed acute renal failure due to tumor progression and was recommended for nephrostomy to relieve obstruction, followed by antitumor treatment. On August 16, 2024, bilateral nephrostomy was performed under local anesthesia, and kidney function gradually improved thereafter. Next-generation sequencing revealed no detectable mutations in *MET*, *KRAS*, *NRAS*, *ROS1*, *BRAF*, or *KIT*. However, the tumor harbored both high-level ERBB2 (HER2) amplification, with an estimated copy number of 74.5, and a pathogenic ERBB2 point mutation (c.2264T>C, p.L755S, exon 19), detected at a high variant allele frequency of 98.36%. Given the patient’s acute renal injury and elevated creatinine levels, traditional chemotherapy was considered unsuitable due to compromised renal function. Genetic testing revealed the presence of HER2 co-alteration, including both HER2 amplification and a HER2 point mutation, suggesting potential sensitivity to HER2-targeted therapy. Therefore, the patient was administered an exploratory treatment regimen consisting of T-DXd (200 mg on Day 1) and toripalimab (240 mg on Day 1), for a total of nine treatment cycles. (**Figure 2**). Imaging assessments (chest and abdominal CT scans) were performed at approximately six-week intervals during the treatment period, specifically on October 2, 2024; November 12, 2024; December 24, 2024; and February 6, 2025. On September 11, 2024, following two cycles (approximately six weeks), a partial response (PR) was observed, with significant tumor shrinkage as assessed by the Response Evaluation Criteria in Solid Tumors (RECIST) guidelines. Subsequent evaluations on October 23, 2024, December 4, 2024, and January 16, 2025, showed stable disease (SD), with no progression but no further significant shrinkage of the tumors (**Figure 3**). As of February 7, 2025, the patient continues maintenance therapy with the same regimen and is being closely monitored for further disease progression. During the treatment period, complete blood count and liver function tests were monitored every three weeks. The patient tolerated the treatment well, with no significant adverse reactions observed.

## Discussion

Current clinical management strategies for cervical mucosal melanoma primarily revolve around surgical intervention, supplemented with radiotherapy, chemotherapy, targeted therapy, and immunotherapy. However, the lack of standardized protocols due to disease rarity and insufficient large-scale clinical studies contributes to its universally poor prognosis. Despite aggressive surgery, postoperative recurrence rates remain alarmingly high, reaching 60%-80% (14). For advanced or recurrent cases, radiotherapy and chemotherapy (e.g., dacarbazine, temozolomide) are often used as adjuvant treatments, but their efficacy remains limited, providing only temporary local control. In recent years, immune checkpoint inhibitors (e.g., PD-1/PD-L1 inhibitors, CTLA-4 inhibitors) and targeted therapies (against BRAF or KIT mutations) have been introduced into clinical practice, with some cases showing prolonged survival. However, the overall response rate remains lower than that observed in cutaneous melanoma (15). Studies suggest that combining immunotherapy with radiotherapy may enhance local control, but large-scale clinical evidence is still lacking (16). Notably, mucosal melanoma exhibits distinct molecular characteristics compared to its cutaneous counterpart, such as a lower BRAF mutation rate and a higher prevalence of NF1 mutations, which restrict the applicability of targeted therapies (17). The 5-year survival rate remains below 15%, with early metastasis and high recurrence rates posing major challenges (18). Therefore, future efforts should focus on exploring comprehensive treatment models based on molecular subtyping and advancing exclusive clinical trials specifically for mucosal melanoma.

T-DXd is the new standard second-line treatment for HER2-positive metastatic breast cancer. In the DESTINY-Breast03 trial, compared to trastuzumab emtansine (T-DM1), T-DXd significantly extended progression-free survival (PFS) (median 28.8 months vs. 6.8 months) and overall survival (OS) (HR 0.33), with manageable safety (10). Additionally, T-DXd is the first ADC approved for HER2-low metastatic breast cancer (IHC 1+ or 2+/ISH negative), particularly effective in hormone receptor-positive or triple-negative breast cancer, and is recommended for use after chemotherapy (19, 20). In gastric cancer/gastroesophageal junction adenocarcinoma, T-DXd is approved for advanced patients previously treated with trastuzumab, with the DESTINY-Gastric01 trial showing significantly superior objective response rates compared to traditional chemotherapy (intravenous irinotecan or paclitaxel) (21). T-DXd is the first targeted therapy approved for HER2-mutant advanced non-small cell lung cancer (NSCLC), demonstrating durable antitumor activity in trials such as DESTINY-Lung02 (22). Although T-DXd has demonstrated significant efficacy in HER2-overexpressing gynecological malignancies such as cervical, endometrial, and ovarian cancers (23), there are currently no direct research reports on the use of T-DXd for the treatment of cervical mucosal melanoma.

This case report is the first to document significant clinical benefit achieved with the combination of toripalimab and T-DXd, in a patient with ERBB2-amplified cervical mucosal malignant melanoma. ERBB2 (the gene encoding HER2) amplification or overexpression drives tumor proliferation and metastasis through constitutive activation of downstream MAPK/PI3K signaling pathways (24). In mucosal melanomas, ERBB2 amplification occurs in 2%-5% of cases and correlates with aggressive behavior and poor prognosis (25). Studies confirms that high-level ERBB2 amplification reliably predicts HER2 protein overexpression, making it an ideal target for antibody-drug conjugates (26–28). As a next-generation HER2-targeted ADC, T-DXd demonstrates unique advantages through its deruxtecan payload, which exhibits potent membrane permeability and bystander effects - enabling sustained antitumor activity even in heterogeneous tumors with coexisting RAS/RAF mutations (29). The observed rapid and durable tumor regression aligns with preclinical evidence showing T-DXd’s robust efficacy in ERBB2-amplified xenograft models.

Mucosal melanomas are characterized by an immunosuppressive tumor microenvironment, where single-agent PD-1 inhibitors achieve response rates below 20% (30). Toripalimab may restore T-cell function by blocking PD-1/PD-L1 axis, while T-DXd potentially enhances antitumor immunity through immunogenic cell death and antigen release (31). This synergistic mechanism is supported by clinical data showing toripalimab combination with antiangiogenic agents (e.g., axitinib) elevates objective response rates to 48.3% in metastatic mucosal melanoma (5). Our findings further validate the feasibility of combining PD-1 inhibition with targeted therapy, potentially mediated by increased tumor-infiltrating CD8^+^ T cells observed post-treatment. Previous studies have demonstrated that tumors with ERBB2 amplification exhibit primary resistance to traditional anti-HER2 monoclonal antibodies (e.g., trastuzumab), while ADCs such as T-DXd can overcome therapeutic limitations posed by HER2 low expression or heterogeneous amplification through efficient delivery of topoisomerase I inhibitors (32, 33). In this case, the application of T-DXd aligns with literature reports. Literature indicates its significant improvement of CD3^+^-CD8^+^ T-cell ratios in neoadjuvant therapy for mucosal melanoma, positively correlating with pathological response rates (5). The combination therapy may create a synergistic “targeted killing + immune activation” effect, particularly beneficial for immunologically weak mucosal melanomas.

Primary cervical mucosal melanoma characterized by diagnostic challenges and lack of standardized treatment protocols (34). This case establishes a biomarker-driven treatment paradigm for this rare subtype through molecular confirmation of ERBB2 amplification, achieving a breakthrough beyond the limitations of traditional chemotherapy or single-agent immunotherapy. Though ERBB2 amplification is uncommon in melanoma, it may serve as a sensitivity marker for ADCs, enabling cross-cancer learning from targeted strategies in breast and lung cancers. While previous studies attributed HER2-targeted drug resistance to ERBB2 mutations or downstream pathway activation, this case suggests that in ERBB2 amplified tumors, ADC-immune checkpoint inhibitor combinations may reverse resistance through: (1) T-DXd-induced immunogenic cell death enhancing PD-1 inhibitor efficacy; (2) Toripalimab-mediated reversal of T-cell exhaustion and tumor microenvironment modulation. These mechanisms align with the cross-tumor synergistic effects observed in studies like JUPITER-02 (35).

This case underscores the necessity of systematic molecular profiling for rare malignancies. Although ERBB2 amplification may coexist with RAS/RAF co-mutations causing resistance to conventional anti-HER2 regimens, T-DXd maintains sensitivity (33). Therefore, routine ERBB2 IHC/FISH and NGS testing is recommended for cervical mucosal melanoma to identify potential beneficiaries. This case presents a promising novel therapeutic strategy for ERBB2-amplified cervical mucosal malignant melanoma, highlighting the value of molecular subtyping in guiding off-label therapies. Future multicenter collaborations should establish precision treatment systems for rare mucosal melanomas while exploring ADC-immunotherapy synergy mechanisms, ultimately improving survival outcomes for these highly aggressive malignancies.

## Data Availability

The original contributions presented in the study are included in the article/Supplementary Material. Further inquiries can be directed to the corresponding author.
